# The Oxytocin Receptor (*OXTR*) Contributes to Prosocial Fund Allocations in the Dictator Game and the Social Value Orientations Task

**DOI:** 10.1371/journal.pone.0005535

**Published:** 2009-05-20

**Authors:** Salomon Israel, Elad Lerer, Idan Shalev, Florina Uzefovsky, Mathias Riebold, Efrat Laiba, Rachel Bachner-Melman, Anat Maril, Gary Bornstein, Ariel Knafo, Richard P. Ebstein

**Affiliations:** 1 Department of Psychology, The Hebrew University of Jerusalem, Jerusalem, Israel; 2 Department of Human Genetics, The Hebrew University of Jerusalem, Jerusalem, Israel; 3 Brain and Behavior Science, The Hebrew University of Jerusalem, Jerusalem, Israel; 4 Center for the Study of Rationality and Interactive Decision Theory, Jerusalem, Israel; 5 S. Herzog Memorial Hospital, Jerusalem, Israel; Claremont Graduate University, United States of America

## Abstract

**Background:**

Economic games observe social decision making in the laboratory that involves real money payoffs. Previously we have shown that allocation of funds in the Dictator Game (DG), a paradigm that illustrates costly altruistic behavior, is partially determined by promoter-region repeat region variants in the arginine vasopressin 1a receptor gene (*AVPR1a*). In the current investigation, the gene encoding the related oxytocin receptor (*OXTR*) was tested for association with the DG and a related paradigm, the Social Values Orientation (SVO) task.

**Methodology/Principal Findings:**

Association (101 male and 102 female students) using a robust-family based test between 15 single tagging SNPs (htSNPs) across the *OXTR* was demonstrated with both the DG and SVO. Three htSNPs across the gene region showed significant association with both of the two games. The most significant association was observed with rs1042778 (p = 0.001). Haplotype analysis also showed significant associations for both DG and SVO. Following permutation test adjustment, significance was observed for 2–5 locus haplotypes (p<0.05). A second sample of 98 female subjects was subsequently and independently recruited to play the dictator game and was genotyped for the three significant SNPs found in the first sample. The rs1042778 SNP was shown to be significant for the second sample as well (p = 0.004, Fisher's exact test).

**Conclusions:**

The demonstration that genetic polymorphisms for the *OXTR* are associated with human prosocial decision making converges with a large body of animal research showing that oxytocin is an important social hormone across vertebrates including *Homo sapiens*. Individual differences in prosocial behavior have been shown by twin studies to have a substantial genetic basis and the current investigation demonstrates that common variants in the oxytocin receptor gene, an important element of mammalian social circuitry, underlie such individual differences.

## Introduction

The nascent field of neuroeconomics has in a short time made major strides in elucidating the neurobiological underpinnings of social decision making. Neuroeconomics seeks to explain the brain mechanisms underlying a broad range of human behaviors exhibited not only by the marketplace but also by the matrix of human relationships that constitute more complex community interactions. An important contribution of neuroeconomics is the demonstration that the classical economic assumption of self-interest cannot explain the richness of human social behavior [1]. Economic games, which observe human decision making in the laboratory and involve the benefits of real outcomes (real money) and quantifiability, provide a more coherent framework to benchmark social behavior. Combined with brain imaging and pharmacological manipulations, economic games have provided critical insights into the neural circuits which drive economic and social decision cognition [2]. In addition, these findings have suggested an evolutionary basis for many of our affective and cognitive biases, particularly other-regarding behavior.

In a seminal study [3] we have shown that the powerful techniques of molecular genetics can also be employed to further clarify the mechanisms by which the social brain formulates economic decisions. We demonstrated that allocation of funds in the Dictator Game (DG) were in part determined by length of the arginine vasopressin 1a (AVPR1a) RS3 promoter region repeat region. Additionally, the length of the RS3 repeat region was correlated with increased amounts of *AVPR1a* mRNA in hippocampal post-mortem specimens. The DG is a simple one-shot, two player game. The first player, or “Dictator”, makes a unilateral decision regarding the split of a fixed sum of money between herself and a second player. The second player, or “Recipient”, must accept the result. Fund allocation in the Dictator Game is considered a real-life test of altruism since it involves a ‘put your money where your mouth is’ decision where giving to another is both costly and not subject to strategic considerations of reciprocity; this is particularly the case when “Dictator” and “Recipient” identities are made anonymous [4]. Moreover, allocations in the DG underscore the universal nature of human altruism since the canonical model - based on self-interest – has been shown to fail in all societies studied [5].

Our first studies of the role of the *AVPR1a* gene in social cognition exemplified by a simple laboratory paradigm converges with a wide range of both animal and human experimentation showing that arginine vasopressin (AVP) and the closely related oxytocin (OT) are key neuropeptides that facilitate social communication, affiliative behaviors and social cognition across mammals [6], [7] . Not only has the role of AVP and OT been independently shown to enhance a range of social behaviors across the class of Mammalia including humans, but the interaction between these two social hormones has also been broadly documented [8]–[10]. OT and AVP have overlapping functions in part mediated by mutual actions on their respective receptors [11]–[15], possibly based on receptor affinities [16]. Moreover, behavioral and tissue binding studies suggest that considerable cross-communication is possible between the functional effects of OT and AVP[14], [17]–[19]. We hypothesized, based on our first findings showing that the length of the *AVPR1a* RS3 promoter region repeat predicted Dictator Game allocations [3], that the *OXTR* gene would also likely modulate other-regarding behavior in this simple laboratory game.

Intriguingly, intranasal application of OT has been shown to increase trusting behavior in humans [20], [21], identification of others' affective states [22] and amygdala responses to emotional faces [23]. Administration of OT also improves social cognition in autism [24]. In addition to their roles in enhancing social behaviors, the AVPR1a and oxytocin receptor (*OXTR*) genes have been shown by us [25], [26] and others [27]–[30] to contribute to autism spectrum disorders (ASD), a behavioral disorder characterized by deficits in social communication and bonding. A recent study by Zak et al.,[31] had subjects indicate transfer amounts for both the DG and the Ultimatum game (where the recipient has the opportunity to refuse the allocation, in which case both players receive zero) after undergoing intranasal administration of OT. Oxytocin significantly increased transfer amounts in the Ultimatum game as compared to placebo, whereas no effect in the DG was observed. An effect the authors attributed to the increased perspective taking in the ultimatum game, since in addition to indicating transfer amounts allocators also had to take into account the threat of punishment.

Genetic association studies for complex phenotypes such as social behavior can be highly sensitive to measurement construct. For example, a meta-analysis reviewing association for a serotonin transporter gene polymorphism (*5-HTTLPR*) and anxiety related traits demonstrated how choice of measurement scale may be a critical factor in moderating phenotypic contours[32]. To this end, we also explored allocation of funds in a second, related paradigm, Social Value Orientations (SVO)[33]. We selected this paradigm since it addresses related, yet distinct aspects of prosocial behavior than the Dictator Game. While the Dictator is a zero-sum game with a fixed pie, the SVO enables individuals to allocate funds with relatively small costs, tapping into a broad motivational orientation that is largely independent of the costs of sharing. Social Value Orientations (SVO)[34], use a set of interdependent payouts to categorize individuals based on two different types of other regarding preferences: concern for the well-being of others, and concern for equality. In SVO experiments, subjects are asked to make choices regarding the distribution of points (which in the present study had real monetary value) between themselves and an ‘other’. Consistent value orientations classify players as prosocial- maximizing joint outcomes, individualistic- maximizing personal outcomes or competitive-maximizing the difference between individual and ‘other’ outcome. In making comparisons between orientations, it is also common to group ‘competitive’ and ‘individualistic’ subjects into a general ‘pro-self’ cohort[34]. We hypothesized that positive associations for both high giving in the Dictator Game and prosociality in the Social Value Orientations would provide additional evidence for the robustness of SNP association beyond a specific, singular measure. Altogether, we tested association between 16 tagging SNPs (htSNPs) across the entire *OXTR* gene region and individual distribution of endowments in these two games. Our selection of all tagging SNPs listed for *OXTR* in HapMap represents an optimal strategy for detecting association across this gene region [35].

## Results

### Fund allocation in Dictator Game and Social Values Orientation Task

**Figure 1:**
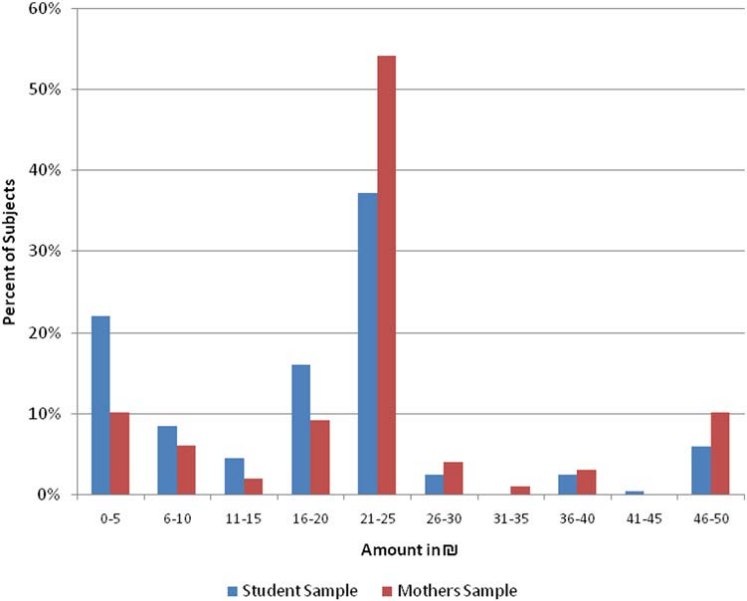
Distribution of Dictator allocations for student and mother samples. Allocation sums by participants grouped in 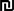 5 increments (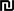 5≈$1.17). For both samples, the modal value of 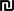 25 was used as the cutoff point to divide participants into low and high allocators.

**Figure 2:**
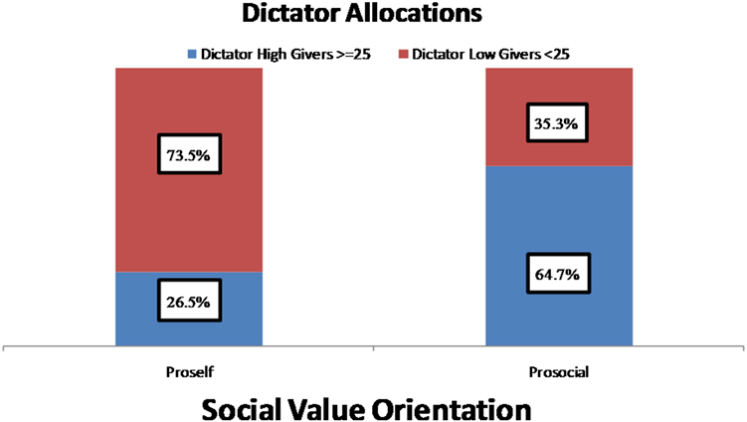
Cross tabulation of Dictator and SVO allocation of funds. Cross tabulation of Dictator giving with Social Value Orientation (SVO). High givers in the DG were significantly more likely to maintain prosocial value orientations as compared to low givers.

Allocation amounts in the DG were dichotomized into low (<25 NIS, or less than 50% of the pie) and high givers (≥25 NIS) [3]; 25 NIS was the modal value in this distribution and was used as the cutoff point to divide participants into low and high allocators (see Figure 1). Altogether, 47% of the participants were designated as high allocators. Based on their responses in the SVO task, participants were categorized into one of three groups: prosocial, proself (made up of both pro-individuals and competitors) and undefined (participants that had less than six responses consistent with any one category); 58% of participants were categorized as prosocial, 36% as proself (which includes 1% competitive and 35% individualistic) and 6% as undefined. Similar to dictator giving, prosocial responses also fell into a bi-modal distribution with a plurality (38%) of participants selecting 9/9 prosocial responses and 24% selecting 0/9. There is considerable similarity between individual allocation of funds in the Dictator Game and the Social Values Orientation (SVO) task (Figure 2, Pearson Chi-Square = 28.18, DF = 1 p<0.0001). In the SVO prosocial group, 64.7% of respondents are also high allocators (≥25 NIS) in the Dictator Game. Among proself SVO individuals, only 26.5% are high allocators in the Dictator Game.

### Association between OXTR htSNPs and fund allocation

**Figure 3:**
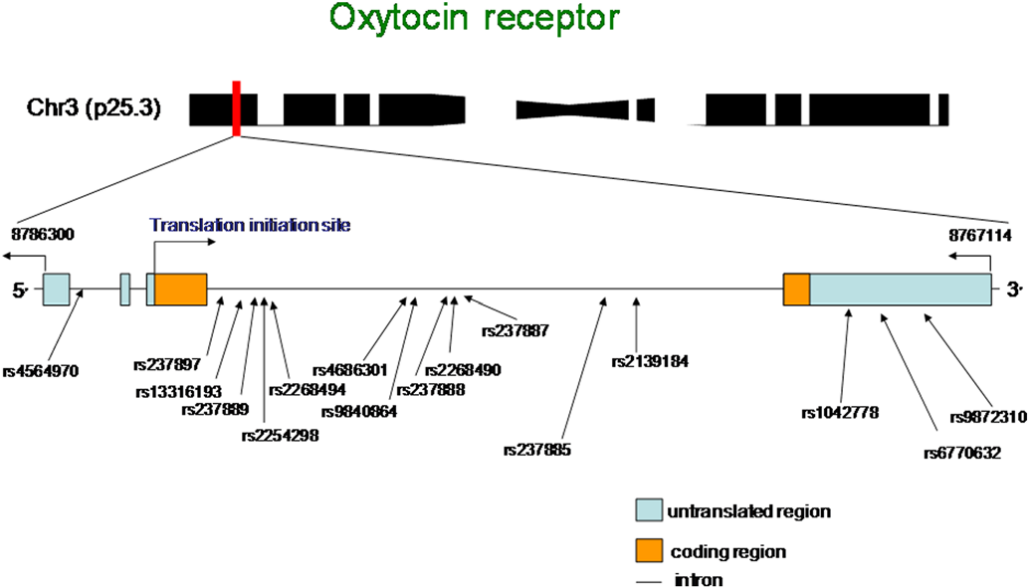
Visual schematic of the oxytocin receptor. Schematic representation of chromosome 3 and the oxytocin receptor (OXTR) gene with the location of the 16 tagging single-nucleotide polymorphisms (SNPs).

**Figure 4:**
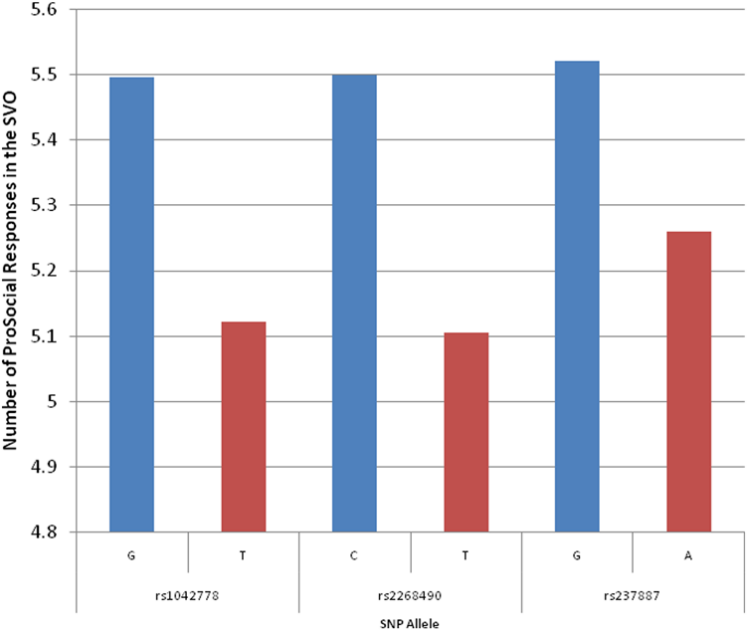
Comparison of SVO Prosocial Responses by OXTR SNPs. Average number of prosocial responses in the Social Value Orientation task categorized by the three most significant OXTR SNPs. A family-based analysis for the three SNPs showed significant association with prosocial value orientations (PBAT dominant model: rs1042778 p = 0.001, rs2268490 p = 0.011, and rs237887 p = 0.005).

All of the SNPs tested were in Hardy-Weinberg Equilibrium (p>0.01). Of the 16 tagging SNPs, one of them, rs2139184, had a very low rate of heterozygosity, and was not included in the analysis. We next examined association using PBAT between 15 single tagging SNPs (htSNPs) across the *OXTR* (Figure 3) and allocation of funds in the Dictator Game and the Social Values Orientation task (Table 1), results are given using PBAT's dominant model, which assumes a dominant mode of inheritance. Of the 15 htSNPs across the gene region, 8 htSNPs showed nominal significant association (before correction for multiple testing) with one or both of the game paradigms The most significant association, which is also robust to Bonferroni correction, was observed with rs1042778 for both the Dictator Game (p = 0.001, p corrected 0.016) and SVO (p = 0.002, p corrected = 0.031). There were also SNPs whose effects appeared to be gender specific e.g. rs237897 (nominally significant in male subjects) and rs9840864 (nominally significant in female subjects). 6 of the 8 htSNPs showed a consistent association for both Dictator Game and SVO allocations. For the three most significant SNPs (rs1042778, rs2268490, and rs237887), an allele-based comparison of the number of prosocial responses for the SVO is displayed in Figure 4.

**Table 1:** Association between OXTR SNPs and allocation of funds in the Dictator Game (high versus low allocators) and SVO

RS number	SNP	Minor allele frequency in sample population	DG Overall	DG Males	DG Females	SVO Overall	SVO Males	SVO Females
rs4564970	C/G	C : 0.13						
rs237897	A/G	A : 0.36		0.0052			0.0019	
rs13316193	C/T	C : 0.27	0.0508					
rs237889	C/T	T : 0.39						
rs2254298	A/G	A : 0.19		0.0455			0.0548	
rs2268494	A/T	A : 0.11						
rs4686301	C/T	T : 0.30						
rs9840864	C/T	C : 0.35			0.0184			0.0357
rs237888	C/T	C : 0.08						
rs2268490	C/T	T : 0.22	0.0111	0.0265	0.0398	0.0083		0.0295
rs237887	A/G	G : 0.49	0.0048	0.0068		0.0250	0.0144	
rs237885	G/T	G : 0.48			0.0194			0.0163
rs1042778	G/T	T : 0.29	0.0011	0.0046		0.0021	0.0048	
rs6770632								
rs9872310								

DG = Dictator Game, categorized as high (> = 25) and low (<25) allocators.

SVO = Social Value Orientation, categorized as prosocial vs proself orientations.

### Haplotype analysis

**Figure 5:**
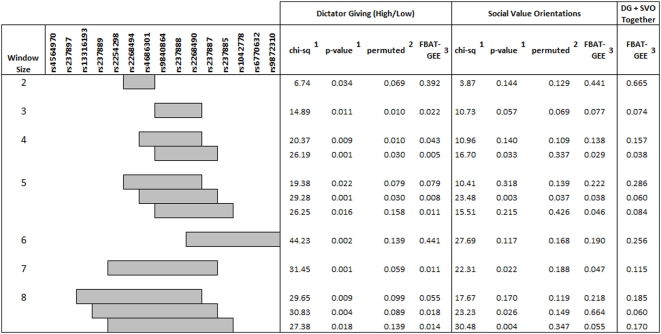
Association between *OXTR* haplotypes and allocation of funds in the Dictator Game (high versus low allocators) and Social Value Orientations. Haplotypes are indicated in outlined blocks. ^1^ UNPHASED global p-value, additive model ^2^ UNPHASED permutation test for the haplotype block ^3^ PBAT multi-variate statistic, dominant model.

Since haplotypes are often more informative than individual SNPs [36], haplotype analysis was carried out. We first used UNPHASED to test associations between 2–8 locus haplotypes since this program features a convenient, automatic sliding window approach that minimizes multiple testing by only testing adjacent SNPs (see FIGURE 5, only significant haplotypes are shown). Haplotype analysis using UNPHASED calculates overall global p values that corrects for multiple testing of individual haplotypes. Global-*P-*values are determined in UNPHASED to compare the maximum observed SNP score in the observed data against each maximum of the SNP score in the permuted data set. For Dictator Giving, significant associations were observed for 2–8 locus haplotypes (global p<0.05), many of which were also significant for the SVO.

We then used the stringent permutation test option provided in the UNPHASED software package to further void spurious results and correct for multiple testing. Following permutation testing, significance was observed for 2–5 locus haplotypes (p<0.05) and approached significance for the 7 and 8 locus haplotypes (permutation test p<0.1). See FIGURE 5, ‘permuted’ column.

Next, to rigorously test the robustness of the associations observed with UNPHASED we employed the multivariate statistic (FBAT-GEE) utilized in the PBAT software package for the haplotype blocks assessed in UNPHASED. Simulation experiments involving quantitative traits show that the multivariate FBAT clearly outperforms permutation tests and individual FBATs with corrections for multiple testing. Lange et al. [37] has proposed a natural extension of the FBAT statistics which are based on the general likelihood-score test approach [38] to multivariate phenotypes based on GEE scores. Without making any assumptions about the distributions of the phenotypes, the multivariate FBAT test allows us to test the null hypothesis that the marker locus is not linked to any genetic locus that has an influence on the selected phenotypes.

When several phenotypes are selected, all phenotypes are tested simultaneously using FBAT-GEE. For FBAT-GEE, both binary and continuous phenotypes can be entered. The program can combine phenotypes with different distributions (e.g. continuous and ordinal). Generally, the FBAT-GEE statistic can handle a moderate amount of any type of multivariate data, including groups of dichotomous phenotypes. This is an attractive methodology, given the increasingly complex traits, such as prosocial behavior, which we have investigated. Notably, all significant (global p<0.05) haplotype blocks that withstood permutation tests in UNPHASED for Dictator Giving and the SVO were also significantly associated using the PBAT multivariate statistic which considers the DG and SVO simultaneously (p<0.05) (see FIGURE 5, FBAT-GEE column) strongly confirming the results observed in UNPHASED. Notably, the 4 locus haplotype block (rs9840864-rs237888-rs2268490-rs237887) is significant for Dictator Giving (FBAT-GEE = 0.005), SVO (FBAT-GEE = 0.028) and when both Dictator Giving and SVO are jointly considered (p = 0.038).

### Second Sample

After completion of the first study discussed above, a second group of 98 female subjects participating in a separate protocol in the psychology department became available, and were recruited to play the dictator game. To limit the conundrum of multiple testing, we used a more stringent criteria for SNP selection in the second sample[39]. Subjects were genotyped solely for the three SNPs found to be significant in both the SVO and Dictator in the first sample (rs2268490, rs237887 and rs1042778).

Dictator giving was considerably higher in the second sample (One-way ANOVA F_1,296_ = 9.934 p = 0.002), with giving amounts on average 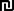
4.97 higher in the second sample (see Figure 1). Using the same dominant model of inheritance as in the first sample, a significant association was observed with the G allele of the rs1042778 SNP and high/low giving in the (p = 0.004, Fisher's exact test). There was also a trend for mean differences in overall dictator giving (F_1,96_ p = 0.07), with TT genotypes allocating on average 18.3 shekels to the ‘other’ and GG+GT genotypes allocating on average 25 shekels to the other, an effect size of approximately 0.5 SD. Notably, SNP rs1042778 was the most significant SNP associated with DG and SVO in the first sample and this association with DG allocations was replicated in the second, independently recruited group of subjects.

## Discussion

This is the second report in our ongoing investigation of the role of common genetic polymorphisms in shaping human altruism as measured in experimental economic games. We have employed a candidate gene approach towards understanding the molecular architecture of altruism and focused our first studies on the AVP – OT pathway, nonapeptides that have been elegantly shown in lower mammals to profoundly impact on so-called ‘social circuits’ [10]. Neural networks employing AVP and OT uniquely influence both social recognition and the expression of appropriate social responses[40]. It is increasingly evident from our own studies [41]–[45] and those of other groups [21], [46]–[54] that these two ‘social hormones’ also contribute to a range of normal human social behaviors.

We had previously shown that the promoter region repeat length of the *AVPR1a* RS3 microsatellite polymorphism contributes to individual differences in Dictator Game allocation of real money and correlates with hippocampal mRNA levels [3]. We now extend these findings and demonstrate that a close, phylogenetically related receptor, OXTR, also impacts on money allocations in laboratory-based economic games that model real life prosocial versus proself behaviors. Additionally, our finding of a significant association for both the Dictator game and SVO, decision tasks that are significantly correlated with one another, provides supporting evidence for the robustness of the current findings.

A previous study by Zak et al. [31] noted a lack of effect of intranasal administration of oxytocin on DG giving, however, in the case of the Ultimatum Game, where participants also have to consider the second player's perspective, oxytocin increased generosity levels. The lack of convergence between the results obtained by sniffing oxytocin and the currently reported results using a genetic association study to establish the neurochemical pathways underlying Dictator Game behavior appear to be at odds. However, it should be considered that the pharmacological approach used by Zak et al [31] and the molecular genetic approach used in the current research represent very different experimental modalities. Perhaps the resolution of these apparent discrepant results awaits a combined pharmacogenetic strategy where subjects (administered oxytocin or placebo) play in the Dictator Game and are stratified by genotype.

We have reported association *OXTR* htSNPs previously in an association study of autism spectrum disorder (ASD) [25]. Notably, three of the SNPs (rs2268490, rs237887 and rs1042778) associated most robustly with the two economic decision tasks reported in the current investigation were also significantly associated with ASD[25]. One of the triad of core deficits in ASD is social communication, and it makes sense that a gene contributing to prosocial behavior in nonclinical populations may also contribute to deficits in social behavior in clinical groups such as ASD. Of particular interest is rs1042778, which is significant both in our prior autism study[25], the 1^st^ sample for both Dictator and SVO and in the second independently recruited DG sample. Interestingly, this SNP is located in the 3′ untranslated region. 3′ untranslated regions (3′ UTRs) often contain several regulatory elements that govern the spatial and temporal expression of an mRNA. [55] Furthermore, several translational regulators are highly conserved and seem to control many different mRNAs. We suggest, based on our observation that this SNP shows significant association with altruism and autism, the possibility that rs1042778 may play an important regulatory role in *OXTR* transcription and translation.

A regulatory role for the intronic (3^rd^ intron) htSNPs (or SNPs in LD with these htSNPs) analyzed in this study should also be considered. Suggestion of a functional character of SNPs located in the 3^rd^ intron is based on the finding of a specific motif of about 10–15 nucleotides close to the middle of the 3^rd^ intron that specifically binds nuclear proteins correlating with the down-regulated state of the gene [56]. Indeed, Mizumoto et al [56] suggested that this intronic element is specifically binding nuclear protein(s) associated with a suppression of *OXTR* gene activity. In addition, to this intronic regulation site, a number of 5′ upstream regulatory sites have been identified for the *OXTR* promoter region [57]. In fact, tagging SNPs analyzed in the current investigation may be detecting signals from other regulatory elements along the OXTR extended gene region.

Although twin studies underscore the importance of the genetic contribution to prosocial behavior [58], [59], untangling the contribution of specific polymorphisms to this and other complex traits is difficult. Complex traits including human altruism are molded by both environment and multiple genes and such phenotypes pose special challenges for genetic analysis due to gene-gene and gene-environment interactions, genetic heterogeneity, low penetrance, and limited statistical power [60]. That variations in these two receptor genes, *OXTR* and *AVPR1a*, jointly contribute to a complex human behavioral phenotype is probably the rule and not the exception. We are interested in testing other elements of the OT-AVP system that we conjecture are also worth examining within the context of a broad candidate gene-neural network approach towards understanding the fine contours of how these two neurohormones mold social behavior in our own species.

## Materials and Methods

### Subjects

The participants in our ongoing studies of personality, primarily college students and their families, were recruited by word of mouth and advertisements on campus, as previously described [45], [61]. They were contacted by telephone and asked to participate in online versions of economic games. 93% of contacted individuals agreed to participate. The sample consisted of 102 men and 101 women whose average age was 26.00 (SD = 3.73). Parents of the student sample were also genotyped to allow for a family based analysis, however parents did not participate in the games.

A second sample of 98 subjects, all mothers, average age 34.47 (SD = 4.76) were recruited to play the Dictator Game. Mothers were part of an ongoing twin study examining the heritability of prosocial behavior[62]; time constraints of their participation precluded playing in the SVO.

### Ethics Statement

All subjects gave informed written consent and the genetic study was approved by the local University and Hospital Internal Review Board and the Israeli Ministry of Health (Genetics Section).

### Dictator Game

In the student sample, subjects were provided with a login and password to an online site and upon entering were provided with instructions for the DG and SVO. Ordering of DG and SVO was randomized and there was no effect of game order on average giving amount or value orientation. In the second sample, subjects played the dictator game at the Hebrew University Social Development lab as part of an ongoing study on genetic and environmental influences on prosocial behavior in early childhood. Game instructions were identical for both groups.

Dictator Game instructions to participants were as follows:

Dear participant,

In this task you will be asked to make a decision which can earn you some money. The task will take place in pairs, in which case one of the participants will be Player A, and the other participant will be Player B. The assignment of Player A and Player B will be done randomly by the computer. You do not know the other participant and will not knowingly meet them in the future.

In this task there are 50 points that Player A must decide how to distribute between himself and Player B. That is, Player A decides how many points he will keep for himself and how many points Player B will receive. For every point that he keeps for himself, Player A will receive 1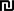 and for every point that he gives to Player ‘B’, Player ‘B’ will receive 1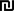.

This completes the task.

Please press the button to continue.

Upon pressing the button, subjects were notified if they were Player ‘A’ or Player ‘B’. In reality, all subjects were selected as Player ‘A’. Upon completing the experiment, subjects were paid via check to their home address.

### Social Value Orientations Task

A measure developed by Van Lange and colleagues (23) to measure social value orientations by using a series of decomposed games, involving choices among combinations of outcomes for oneself and for another person, was adapted in this study. In the current study, instead of assigning hypothetical points to the participant, each point had real monetary value, as described to participants in the following instructions:

Dear Participant,

In this task you will be partnered with a random additional person whom which we will call the “other”. You do not know this person and will not knowingly meet them in the future. You will be asked to make a number of decisions in which you will be required to select between three options ‘A’ ‘B’ or ‘C’. Your selection will provide points for you and for the “other”. At the completion of the task we will sum the number of points you have accumulated for yourself and for the other and will pay you 1 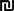 for each 100 points you have collected. Similarly, we will pay the “other” 1 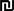 for each 100 points as well.

See Table 2 for an example of the task.

**Table 2:** Social Value Orientation Example Task

	A	B	C
You Receive	500	500	550
Other Receives	100	500	300

Based on this example, if you were to select:

‘A’ you would receive 500 points = 5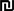 and the “other” would receive 100 points = to 1
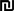

‘B’ you would receive 500 points = 5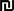 and the “other” would receive 500 points = to 5
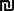

‘C’ you would receive 550 points = 5.5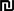 and the “other” would receive 300 points = to 3
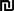

As you can see, your decisions affect both the number of points you receive and the number of points the “other” receives. Before you begin your selections, realize that there are no right or wrong answers. Pick the selections that you prefer for whatever reason. As such, remember that the points have a real money value: 100 points are equal to 1
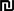

Payment will transpire according to the aforementioned rules and will be transferred to via check to you and the “other” a few weeks after completion of the experiment.

The “other” does not have the opportunity to identify you. Game results are recorded in a way to assure anonymity.

You will only have one opportunity to make your selections. Please weigh your decisions carefully; you will not have an opportunity to go back.

### Genotyping

The *OXTR* was genotyped as previously described [25]. DNA was extracted by Master Pure kit (Epicentre, Madison, WI, USA). Genotyping of all SNPs was performed using the SNaPshot Method (Applied BioSystems, Foster City, CA, USA) which relies upon the extension of a primer immediately adjacent to the SNP using fluorescently labeled ddNTPs. The fluorescently labeled extension primers can then be visualized by electrophoresis on a capillary ABI PRISM 310 automated sequencer. *OXTR* SNP regions were amplified using the pairs of primers listed in Table 3. PCR cycling conditions in the SNaPshot Method were as follows: samples were initially heated at 94°C for 5 min followed by 35 cycles of 94°C (30 s), 55°C (30 s) 72°C (90 s) and a final extension step of 72°C for 5 min. After the first PCR cycle, the PCR product was cleaned with ExoSAP for 37°C for 30 min and then at 80°C for 15 min. The conditions for the second PCR were as follows: 96°C (10 s), 50°C (5 s) and 60°C (30 s) for 25 cycles. The second PCR product was cleaned using shrimp alkaline phosphatase (SAP) initially at 37°C for 1 h followed by 72°C for 15 min.

**Table 3:** Primers used for genotyping of OXTR tagged SNPs

PCR I (5′ to 3′) Forward	PCR I (5′ to 3′) Reverse	SNP	PCR II (primer extension 5′ to 3′)
TTGTAATTCTAATGCACCCTCA	AAAGTTGAAAGATCCAAAGAGTAAA	rs237887	AGCTTTGCAATGAGGTAG
		rs2264890	CAGAAAGACACTGTTTTG
		rs237888	(T)_24_ATTGTGACCAATAACTGT
CCCAGAGGTCTGTGGGTGTA	GTCAGGGAGGAGCTGTTCTG	rs2268494	(T)_12_GACCACACGGTCCCACAT
		rs2254298	(T)_6_AAGAAGCCCCGCAAACTG
		rs237889	(T)_6_GCAAAGACAGCAAGGCCA
		rs13316193	(T)_36_CGTGGAGGACGGGAATGC
ATTGTCTCGGTGCCATTTGT	CCATCAGAAAGAATAAAATAGGAA	rs9872310	TCAATTGACCGTAAGTAT
		rs6770632	(T)_12_CCTTCTTTAATTTCTTTC
AGAGCTGCCTGCCAAATG	GACCCCCGGAGAAGGTGCT	rs9840864	(T)_12_CAAATGTGCAAAGGCCAG
		rs4686301	(T)_30_CAGCCACAATGATGTCAG
AGGAGAGTGCCCAAACCTCT	GCATCTTTGGGAATCAGCTC	rs2139184	(T)_6_GCTATCACGACCATGTGC
		rs237885	(T)_6_GATGCAGACATCTTGTGG
AAGGGAGGGTCAAAATCAGC	GTTAGACGGGGAAGGACCAG	rs4564970	(T)_18_CCCTCAGCATATCCACCT
TGGGTTCAGGGTGGTAGAAG	AGGCTGTGCTGGCATAAGTG	rs1042778	(T)_12_TGAAGCCACCCCAAGGAG
CCTCCCCCTCAAACTTGAAT	CCAAGGGAGAGGTGAAGACA	rs237897	(T)_36_TCCATAAGCCTGCCCACC

All tagging SNPs listed in HapMap and selected using the Haploview program http://www.broad.mit.edu/mpg/haploview/ were genotyped. Most association studies rely on the use of surrogate markers, single-nucleotide polymorphisms (SNPs) being the most suitable due to their abundance and ease of scoring (see discussion in [35]. SNP marker selection is aimed to increase the chances that at least one typed SNP would be in linkage disequilibrium (LD) with the disease causative variant, while at the same time controlling the cost of the study in terms of the number of markers genotyped and sampled. Empirical studies reporting block-like segments in the genome with high LD and low haplotype diversity have motivated a marker selection strategy whereby subsets of SNPs that ‘tag’ the common haplotypes of a region are picked for genotyping, avoiding typing redundant SNPs.

### Statistics

We tested for association between *OXTR* polymorphisms and money allocations in both the Dictator Game and Social Values Orientation using the ETDT, a logistic based variant of the transmission disequilibrium test (TDT) which assesses for association (and linkage) without the confounding effect of population stratification. The TDT, in its simplest version, compares, for one allele, the number of times this allele is transmitted to the number of times where this allele is not-transmitted to an affected offspring. Note that only heterozygous parents are informative. This approach can be extended to haplotypes. For the second sample, a Fisher's exact test was used to examine the significance of the association (contingency) between genotypes and high/low giving amounts.

### UNPHASED [63], [64]

The various tests we used are implemented in the program *UNPHASED* (http://www.rfcgr.mrc.ac.uk/~fdudbrid/software/unphased/) [63], a suite of programs for association analysis of multilocus haplotypes from unphased genotype data. Permutation test correction was performed using 1000 iterations (random permutations) and applied to correction of global P-values (the P-value as an overall test of each block of haplotypes). In nuclear families, the permutations are generated by randomizing the transmission status of the parental haplotypes. In unrelated subjects, the trait values are randomly shuffled between subjects.

In each permutation, the minimum P-value is compared to the minimum P-value over all the analyses in the original data. This allows for multiple- testing corrections over all tests performed in a run. This procedure corrects for multiple testing but accounts for correlation between markers, hence is less conservative than a Bonferroni correction, which is appropriate for independent tests such as unlinked markers.

### PBAT (Helix Tree)

The PBAT software package provides a unique set of tools for complex family-based association analysis at a genome-wide level [65], [66]. PBAT can handle nuclear families with missing parental genotypes, extended pedigrees with missing genotypic information, analysis of single nucleotide polymorphisms (SNPs), haplotype analysis, quantitative traits, multivariate/longitudinal data and time to onset phenotypes. The data analysis can be adjusted for covariates and gene/environment interactions. Haplotype-based features include sliding windows and the reconstruction of the haplotypes of the probands.

The family-based association test (FBAT) statistic [38] comprises a linear combination of observed offspring genotypes and traits. We used the HelixTree version of PBAT (http://www.goldenhelix.com/pharmhelixtreefeatures.html). The test statistic is defined as: *S* = ∑*_ij_ T_ij_X_ij_*, where *X_ij_* denotes a coding of the marker genotype of the *j* ^th^ offspring in family *i*. The coded trait of the *j* ^th^ offspring in the *i* ^th^ family is defined by *T_ij_*. In general, the trait will be mean-centered. The test statistic, a score function, in large samples, is defined as: *Z* = ∑*_ij_ T_ij_*(*X_ij_*−*E*(*X_ij_*))/*Var*(*S*)^1/2^∼*N*(0,1), where *E*(*X_ij_*) is the expected value of the coding function for the offspring genotype, conditional on the parental genotype and parental/offspring phenotypes, which are assumed to follow Mendelian segregation under the null hypothesis. This methodology extends readily to scenarios in which the parental genotypes are not known, using the approach of Rabinowitz and Laird [67]. The FBAT statistic has an additional advantage in that it is robust against population admixture and stratification [67].
